# Impact of smoking on the outcomes of minimally invasive direct coronary artery bypass

**DOI:** 10.1186/s13019-023-02104-9

**Published:** 2023-01-20

**Authors:** Youssef Shahin, Ján Gofus, Jan Harrer, Zdeněk Šorm, Martin Voborník, Eva Čermáková, Petr Smolák, Jan Vojáček

**Affiliations:** 1 Department of Cardiac Surgery, Faculty of Medicine and University Hospital in Hradec Kralove, Charles University, Hradec Kralove, Czech Republic; 2 Department of Medical Biophysics, Faculty of Medicine in Hradec Kralove, Charles University, Hradec Kralove, Czech Republic

**Keywords:** Tobacco smoking, Coronary artery bypass grafting, Minimally invasive surgery, Postoperative complications

## Abstract

**Background:**

Tobacco smoking has been associated with an increased risk of complications after conventional coronary surgery. However, the impact of smoking on the risk of postoperative complications in minimally invasive coronary surgery is yet to be studied. We aimed to analyze the impact of the preoperative smoking status on the short- and long-term outcomes of minimally invasive direct coronary artery bypass grafting (MIDCAB) in the context of isolated surgical revascularization or in association with percutaneous coronary intervention.

**Methods:**

This was a retrospective observational study of all patients undergoing MIDCAB at our institution between 2006 and 2020. Patients were divided into three groups: active smokers, ex-smokers who have quit smoking for at least 1 month before surgery, and non-smokers. The groups were compared using conventional statistical methods. Multivariate analysis was then performed where significant differences were found to eliminate bias.

**Results:**

Throughout the study period, 541 patients underwent MIDCAB, of which 135 (25%) were active smokers, 183 (34%) were ex-smokers, and 223 (41%) were non-smokers. Smokers presented for surgery at a younger age (p < 0.0001), more frequently with a history of myocardial infarction (p < 0.001), peripheral artery disease (p < 0.001) and chronic obstructive pulmonary disease (p < 0.0001). Using multivariate analysis, active smoking was determined to be a significant risk factor for the need of urgent revascularization (odds ratio 2.36 [1.00–5.56], p = 0.049) and the composite of pulmonary complications (including pneumothorax, respiratory infection, respiratory dysfunction, subcutaneous emphysema and exacerbation of chronic obstructive pulmonary disease; odds ratio 2.84 [1.64–4.94], p < 0.001). Preoperative smoking status did not influence the long-term survival (p = 0.83).

**Conclusions:**

In our study, active smokers presented for MIDCAB at a younger age and more often with signs of atherosclerotic disease (history of myocardial infarction and peripheral artery disease). Active smoking was found to be the most significant risk factor for postoperative pulmonary complications, and is also associated with a more frequent need for urgent surgery at diagnosis. Long-term postoperative survival is not affected by the preoperative smoking status.

**Supplementary Information:**

The online version contains supplementary material available at 10.1186/s13019-023-02104-9.

## Introduction

Surgical revascularization of the myocardium has been the standard of care in patients with severe coronary artery disease for decades. Coronary surgery has been influenced by the trend towards minimally invasive approaches while preserving excellent patient outcomes. Minimally invasive direct coronary artery bypass grafting (MIDCAB) provides off-pump surgical revascularization of the left anterior descending artery (LAD) with the use of left internal mammary artery graft (LIMA) through a left anterior short thoracotomy. It has kept its popularity throughout the last two decades owing to the lower risk of complications associated with the use of extracorporeal circulation, as well as to the added benefit of its minimally invasive and less traumatic surgical approach [1].

Among a wide scale of factors influencing the outcomes of coronary surgery, tobacco smoking plays an important role. The impact of the smoking status on the outcomes of conventional bypass surgery has been extensively researched, exhibiting an association with an increased risk of several postoperative complications, which include pulmonary and wound healing complications, in-hospital and long-term mortality, renal impairment, strokes, and a longer post-operative stay in the ICU [2–6], Arabaci et al. [6] was able to prove better pulmonary functions in non-smokers post-operatively compared to smokers. Furthermore, evidence suggests that preoperative smoking cessation at least one month before coronary surgery is also associated with better post-operative outcomes [7]. These include a lesser need for inotropic support postoperatively, and a lower rate of pulmonary, neurological, and wound healing complications [8, 9]. However, none of the previous studies have assessed this in the setting of minimally invasive coronary surgery. Therefore, we aimed to analyze the impact of smoking status on the short- and long-term postoperative outcomes in patients undergoing MIDCAB at our institution.

## Methods

This was a retrospective observational cohort study of all patients undergoing MIDCAB at our institution between 2006 and 2020. The perioperative data were collected from patient in-hospital records and manually entered into tables. Long-term data on mortality were provided by the Institute of Health Information and Statistics of the Czech Republic, which is an official institution of the Czech Ministry of Health. The closing date for long-term survival analysis was 30th of December 2020. While the observed long-term outcomes were taken only from the compulsory national registry, we considered the long-term follow-up 100% complete.

Patients were divided into three groups: non-smokers, ex-smokers and smokers. Non-smokers were the patients who had never smoked on a regular basis. Ex-smokers had stopped smoking at least one month prior to surgery. Smokers were the patients who were actively smoking until the surgery or had stopped smoking less than one month prior. The groups were afterwards compared in terms of preoperative cohort characteristics, perioperative outcomes and long-term survival rates.

Pulmonary complications were analysed separately as well as a composite outcome including pneumothorax, respiratory infection, respiratory dysfunction (defined as hypoxemia in arterial blood gas analyses), subcutaneous emphysema and exacerbation of chronic obstructive pulmonary disease (COPD). Urgent surgery was defined in accordance with EuroSCORE II as a case where the patient was either not electively admitted for surgery, required an intervention or surgery at the time of admission, or could not be sent home for medical reasons [10]. Hybrid revascularization was defined as a combined approach in the treatment of multi-vessel coronary disease composed of surgical revascularization of LAD, and percutaneous intervention of other affected coronary vessels, regardless of the timing. However, in certain cases where PCI was not feasible for the other affected coronary vessels, palliative revascularization of the LAD using isolated MIDCAB was performed.

### Surgical technique

Patients were prepared in a supine position. The left chest was elevated approximately to an angle of 20 to 30 degrees. Induction of general anaesthesia was performed conventionally. A double-lumen endotracheal intubation or blocker was routinely used to maintain a single-lung ventilation. The skin incision was eight to twelve centimetres long and was located along the fourth or fifth intercostal space (in men under the left nipple and in women in the skin fold under the left breast). LIMA was harvested under direct vision. The anastomosis of LIMA to LAD was constructed on the beating heart using standard off-pump techniques [11]. Chest drainage was introduced and the thoracotomy was closed.

### Statistical analysis

All the calculations were conducted through NCSS 2019 Statistical Software (2019, Kaysville, Utah, USA). The presented data are shown as categorical or continuous variables. The categorical variables are expressed as numbers and percentages. The continuous data are expressed as medians and interquartile ranges as long as we were unable to prove normal value distribution in most of the variables. The influence of smoking status on the outcomes was tested by Pearson’s Chi-square test for categorical variables, and Kruskal–Wallis nonparametric analysis of variance with post-hoc Dunn‘s test with Bonferroni modification for continuous variables. In all performed tests, a p value of ≤ 0.05 was considered statistically significant.

In statistically significant results, we afterwards performed a multivariate analysis in order to clarify the influence of smoking status on our findings and to detect confounding variables. We utilized the multiple regression analysis for continuous variables and the logistic regression for categorical data. The comparisons were primarily adjusted for all preoperative variables, thereafter the algorithm provided a reduced model with a limited number of input covariates with the best predictability. Hereby the models with the highest predictability are presented. The quality of statistical models was assessed by the coefficient of determination (Model R^2^) and p value of the analysis of deviance.

In the ex-smokers group, we attempted to find a potential cut-off time interval before surgery for smoking cessation to achieve its maximum benefit, with respect to the prevalence of the composite of postoperative pulmonary complications. The group was further divided into two subgroups based on the interval between smoking cessation and surgery and compared. The analyses were performed for the cut-off values of 6, 12, 24, 36, 60, 120, 180 and 240 months interval.

The long-term all-cause postoperative mortality and the composite of cardiac and cerebrovascular mortality were tested by Kaplan–Meier analysis and compared by the log-rank test. The potential influence of preoperative covariates on the long-term all-cause mortality was assessed by the multivariate Cox proportional hazard analysis. Primarily, all preoperative covariates were included in the model and, thereafter, a reduced model with selected number of covariates with the best predictability was provided.

## Results

Throughout the study period, 541 patients underwent MIDCAB at our institution. The cohort included 135 (25%) active smokers, 183 (34%) ex-smokers and 223 (41%) non-smokers. The study groups differed in several preoperative covariates (see Table 1). The smokers presented for surgery at younger age, more frequently with a history of myocardial infarction, peripheral artery disease, and COPD. They had the lowest level of creatinine and EuroSCORE II value, and they were found to be least symptomatic with regard to chest pains. The smallest proportion of females was found in the smokers group. The ex-smokers presented also very often with a history of myocardial infarction and peripheral artery disease. They had the highest prevalence of previous percutaneous coronary intervention and had the highest prevalence of diabetes of the three groups. Non-smokers had the smallest body surface area and the highest ejection fraction of the left ventricle.

**Table 1:** Preoperative cohort characteristics

Variable	Smokers (n = 135)		Non-smokers (n = 223)		Ex-smokers (n = 183)		p value
	Median	(1Q; 3Q)	Median	(1Q; 3Q)	Median	(1Q; 3Q)	
Weight [kg]	**82**	**(72; 94)**	**80**	**(71; 92)**	**85**	**(74; 96)**	**0.049**
Heigth [cm]	**174**	**(168; 178)**	**170**	**(161; 176)**	**172**	**(167; 175)**	** < 0.001**
Age [years]	**58.7**	**(52.9; 66.0)**	**69.2**	**(61.1; 76.2)**	**66.5**	**(58.8; 73.0)**	** < 0.0001**
BMI [kg· m^−2^]	27.3	(24.6; 30.5)	27.9	(25.6; 31.2)	28.4	(25.8; 31.7)	0.05
BSA [m^2^]	**1.96**	**(1.83; 2.12)**	**1.91**	**(1.79; 2.06)**	**1.98**	**(1.84; 2.11)**	**0.01**
Creatinin [μmol/l]	**84.0**	**(70; 97)**	**88.0**	**(75; 103)**	**87.0**	**(76; 105)**	**0.03**
LVEF [%]	**59**	**(45; 65)**	**60**	**(55; 65)**	**55**	**(45; 65)**	** < 0.001**
EuroSCORE II [%]	**0.98**	**(0.69; 1.75)**	**1.23**	**(0.82; 2.09)**	**1.14**	**(0.75; 2.51)**	**0.02**

Variable	Number	Percentage	Number	Percentage	Number	Percentage	p value
Female sex [n, %]	**16**	**(11.9)**	**80**	**(35.9)**	**29**	**(15.9)**	** < 0.0001**
Diabetes [n, %]	**34**	**(25.2)**	**79**	**(35.4)**	**69**	**(37.7)**	**0.049**
Hypertension [n, %]	99	(73.3)	186	(83.4)	139	(76.0)	0.05
Renal Failure [n, %]	5	(3.7)	8	(3.6)	8	(4.4)	0.91
Dyslipidemia [n, %]	77	(57.0)	148	(66.4)	126	(68.9)	0.08
Cardiac arrhythmias [n, %]:							0.66
Atrial Fibrillation/Flutter	6	(4.4)	15	(6.7)	11	(6.0)	
Pacemaker	1	(0.7)	6	(2.7)	3	(1.6)	
Cerebral ATS [n, %]	8	(5.9)	13	(5.8)	15	(8.2)	0.59
PAD [n, %]	**27**	**(20.0)**	**15**	**(6.7)**	**35**	**(19.1)**	** < 0.001**
COPD [n, %]	**35**	**(25.9)**	**12**	**(5.4)**	**28**	**(15.3)**	** < 0.0001**
Stroke [n, %]	10	(7.4)	18	(8.1)	22	(12.0)	0.27
CCS class [n, %]:							**0.02**
I	**57**	**(42.5)**	**60**	**(27.0)**	**66**	**(36.1)**	
II	**33**	**(24.6)**	**69**	**(31.1)**	**60**	**(32.8)**	
III	**23**	**(17.2)**	**55**	**(24.8)**	**40**	**(21.9)**	
IV	**21**	**(15.7)**	**38**	**(17.1)**	**17**	**(9.3)**	
NYHA class [n, %]:							0.09
I	72	(53.7)	85	(38.3)	75	(41.0)	
II	46	(34.3)	87	(39.2)	77	(42.1)	
III	12	(9.0)	40	(18.0)	25	(13.7)	
IV	4	(3.0)	10	(4.5)	6	(3.3)	
History of AMI [n, %]	**77**	**(57.0)**	**95**	**(42.6)**	**111**	**(60.7)**	** < 0.001**
Previous Cardiac Surgery [n, %]	11	(8.2)	21	(9.4)	11	(6.0)	0.45
Extent of Coronary Disease [n, %]:							0.1
1-vessel	70	(51.9)	115	(51.6)	73	(39.9)	
2-vessel	43	(31.9)	67	(30.0)	64	(35.0)	
3-vessel	22	(16.3)	41	(18.4)	46	(25.1)	
Previous PCI [n, %]	**43**	**(31.9)**	**56**	**(25.1)**	**72**	**(39.3)**	**0.009**
Severe heart valve disease [n, %]	17	(12.6)	28	(12.6)	23	(12.6)	1
Intravenous nitrates preoperatively [n, %]	4	(3.0)	4	(1.8)	5	(2.7)	0.7
Anticoagulation preoperatively [n, %]	37	(27.4)	46	(20.6)	31	(16.9)	0.08

The influence of smoking status on the outcomes was tested by Pearson’s Chi-square test for categorical variables, and Kruskal–Wallis nonparametric analysis of variance with post-hoc Dunn ‘s test with Bonferroni modification for continuous variables

Values highlighted in bold were found to be statistically significant

*1Q* first quartile; *3Q* third quartile; *AMI* acute myocardial infarction, *BMI* body mass index; *BSA* body surface area; *CCS* Canadian Cardiovascular Society classification of chest angina; *Cerebral ATS* cerebral atherosclerosis defined as a radiologically proven significant stenosis of cardinal cerebral vessels; *COPD* chronic obstructive pulmonary disease; *EuroSCORE II* risk model for the calculation of the risk of 30-day mortality after cardiac surgery; *LVEF* left ventricular ejection fraction; *NYHA* New York Heart Association classification of dyspnea; *PAD* peripheral artery disease; *PCI* percutaneous coronary intervention; Severe heart valve disease—defined as higher than moderate grade of any heart valve regurgitation/stenosis

Smokers had the highest incidence of indications for urgent revascularization. Subcutaneous emphysema, exacerbation of COPD, and the composite of pulmonary complications were mostly seen in the smokers group. On the margin of statistical significance, we observed also higher incidence of pneumothorax, respiratory dysfunction, and neurological complications in this group. Ex-smokers underwent MIDCAB as a part of a hybrid procedure most frequently. Non-smokers had the longest hospital stay and the highest incidence of wound healing complications. Only four patients died in the short-term postoperatively, which did not provide enough substrate for further statistical evaluation of this outcome. For the complete list of perioperative outcomes see Table 2.

**Table 2:** Perioperative outcomes

Variable	Smokers (n = 135)		Non-smokers (n = 223)		Ex-smokers (n = 183)		p value
	Median	(1Q; 3Q)	Median	(1Q; 3Q)	Median	(1Q; 3Q)	
Operation time [min]	150	(135; 170)	155	(138; 180)	160	(135; 180)	0.2
Artificial ventilation time [hours]	6.7	(4.7; 10.0)	7.6	(5.6; 10.3)	7	(5.0; 9.3)	0.09
24 h Blood loss [ml]	500	(350; 650)	450	(350; 650)	450	(300; 550)	0.14
ICU stay [hours]	24.2	(21.2; 45.8)	24.1	(20.9; 46.0)	23.5	(19.7; 46.8)	0.87
Hospital stay [days]	**8**	**(7.0; 9.0)**	**8.6**	**(7.4; 11.0)**	**8**	**(7.0; 10.0)**	**0.002**

Variable	Number	Percentage	Number	Percentage	Number	Percentage	p value
Urgent surgery [n, %]	**16**	**(11.9)**	**11**	**(4.9)**	**4**	**(2.2)**	**0.002**
Paliative revascularization [n, %]	46	(34.1)	75	(33.6)	66	(36.1)	0.87
Hybrid revascularization [n, %]	**14**	**(10.4)**	**27**	**(12.1)**	**37**	**(20.2)**	**0.02**
Conversion to sternotomy [n, %]	1	(0.7)	9	(4.0)	4	(2.2)	0.15
Reintubation [n, %]	5	(3.7)	3	(1.4)	3	(1.7)	0.27
Catecholamines for > 24 h [n, %]	53	(39.3)	73	(32.7)	70	(38.3)	0.36
Inotropes [n, %]	18	(13.3)	39	(17.5)	25	(13.7)	0.45
Transfusion need [n, %]	17	(12.6)	34	(15.3)	19	(10.4)	0.34
Revision for bleeding [n, %]	6	(4.5)	8	(3.6)	4	(2.2)	0.51
Postoperative infarction [n, %]	2	(1.5)	6	(2.7)	1	(0.6)	0.24
Pleural effusion [n, %]	38	(28.2)	68	(30.5)	45	(24.6)	0.42
Pneumothorax [n, %]	8	(5.9)	7	(3.1)	2	(1.1)	0.05
Subcutaneous emphysema [n, %]	**21**	**(18.9)**	**9**	**(4.8)**	**13**	**(9.0)**	** < 0.001**
Respiratory infection [n, %]	11	(8.2)	11	(4.9)	5	(2.7)	0.09
Respiratory dysfunction [n, %]	18	(13.3)	13	(5.8)	20	(10.9)	0.05
Wound healing complication [n, %]	**1**	**(0.7)**	**14**	**(6.3)**	**9**	**(4.9)**	**0.04**
Neurological complication [n, %]	3	(2.2)	1	(0.45)	0	(0.0)	0.06
Renal Complication [n, %]	4	(3.0)	7	(3.1)	6	(3.3)	0.99
SIRS [n, %]	1	(0.7)	4	(1.8)	1	(0.6)	0.44
Atrial fibrillation postoperatively [n, %]	20	(14.8)	56	(25.1)	38	(20.8)	0.07
Gastrointestinal complication [n, %]	4	(3.0)	8	(3.6)	8	(4.4)	0.8
COPD exacerbation [n, %]	**5**	**(3.7)**	**0**	**(0.0)**	**3**	**(1.6)**	**0.02**
Composite of pulmonary complications [n, %]	**42**	**(31.1)**	**25**	**(11.2)**	**36**	**(19.7)**	** < 0.0001**
Early mortality [n, %]	0	(0.0)	3	(1.4)	1	(0.6)	0.33

The influence of smoking status on the outcomes was tested by Pearson’s Chi-square test for categorical variables, and Kruskal–Wallis nonparametric analysis of variance with post-hoc Dunn‘s test with Bonferroni modification for continuous variables

Values highlighted in bold were found to be statistically significant

*1Q* first quartile; *3Q* third quartile; *24 h* twenty-four hour; *COPD* chronic obstructive pulmonary disease; *ICU* intensive care unit; *SIRS* systemic inflammatory response syndrome

**Fig. 1:**
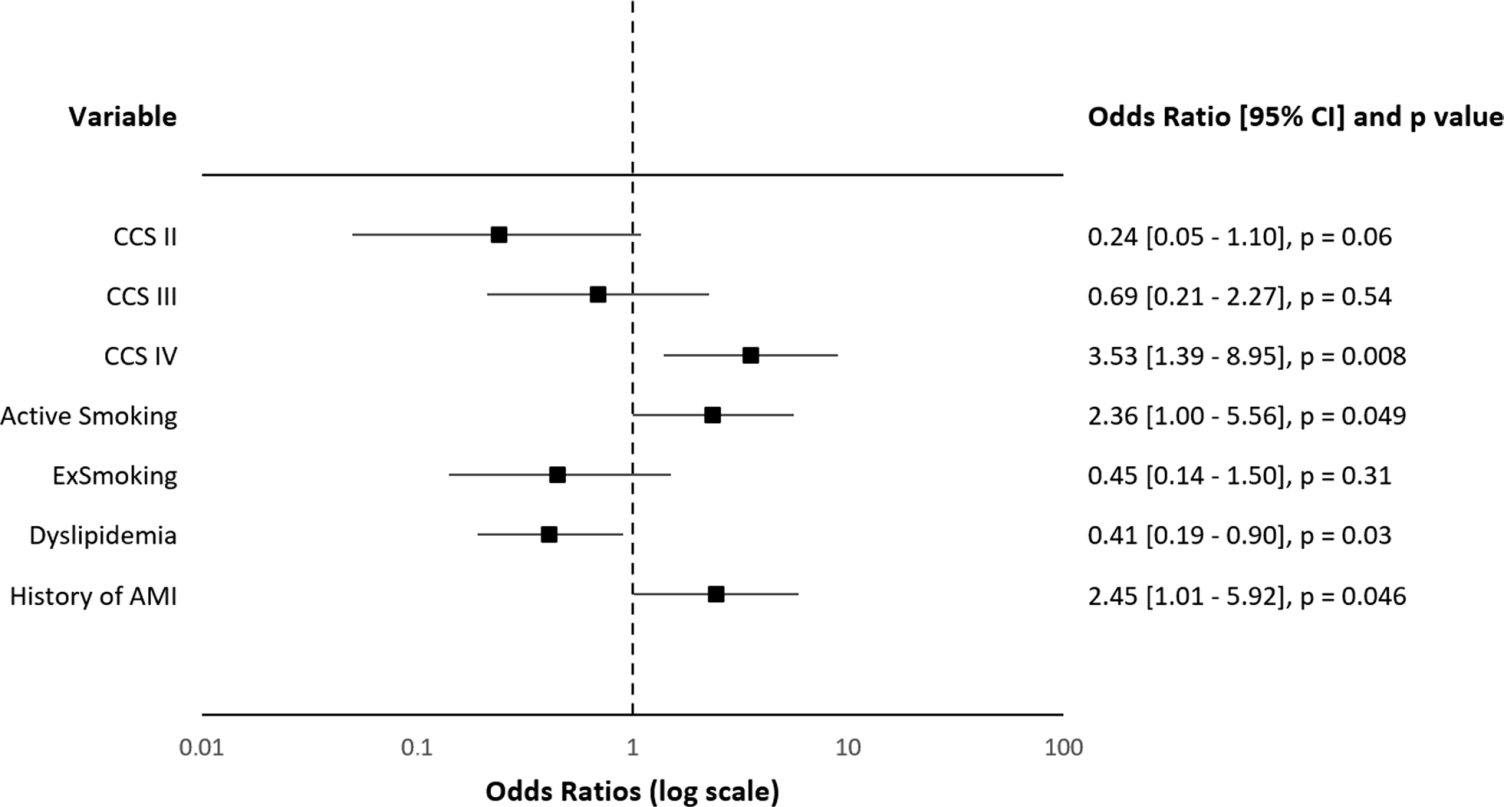
Logistic Regression Analysis for the Need of Urgent Surgery. Model R^2^ = 0.1806; Analysis of deviance for the whole model—p < 0.0001. AMI: acute myocardial infarction; CCS: Canadian Cardiovascular Society classification of chest angina; CI: confidence interval

**Fig. 2:**
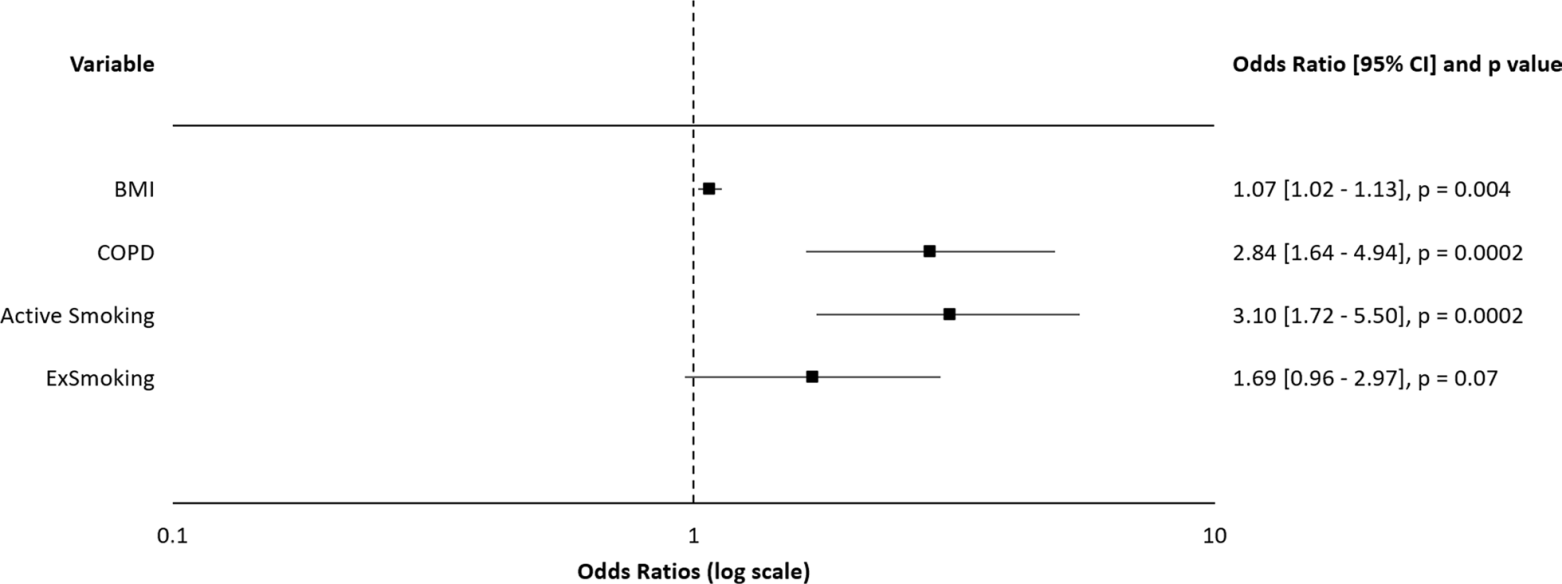
Logistic regression analysis for the coombined endpoint of pulmonary complications. Model R^2^ = 0.0814; Analysis of deviance for the whole model—p < 0.0001. BMI: body mass index; CI: confidence interval; COPD: chronic obstructive pulmonary disease

According to the multivariate logistic regression, active smoking was determined to be a risk factor for the need of urgent revascularization (see Fig. 1) together with the history of myocardial infarction, chest pain at rest, and treated dyslipidemia (as a protective factor). Active smoking was the most significant risk factor for the composite of postoperative pulmonary complications, followed by COPD and body mass index (see Fig. 2). A similar trend was observed with the incidence of subcutaneous emphysema, where the significant risk factors were active smoking and the presence of COPD (see Additional file 1: Figure S1). Subcutaneous emphysema was in some cases concurrent with pneumothorax but often independent of other pulmonary pathologies. Ex-smokers were no longer associated with a significantly higher risk of the abovementioned complications. We were unable to define a cut-off interval before surgery for the best benefit of preoperative smoking cessation with regard to the prevalence of the composite of postoperative pulmonary complications (see Additional file 1: Table S1). Hybrid treatment (p = 0.08), the length of hospital stay (p = 0.58), and the risk of wound healing complications (p = 0.17) were not influenced by the active smoking but by other preoperative covariates (see Additional file 1: Figs. S2–4).

**Fig. 3:**
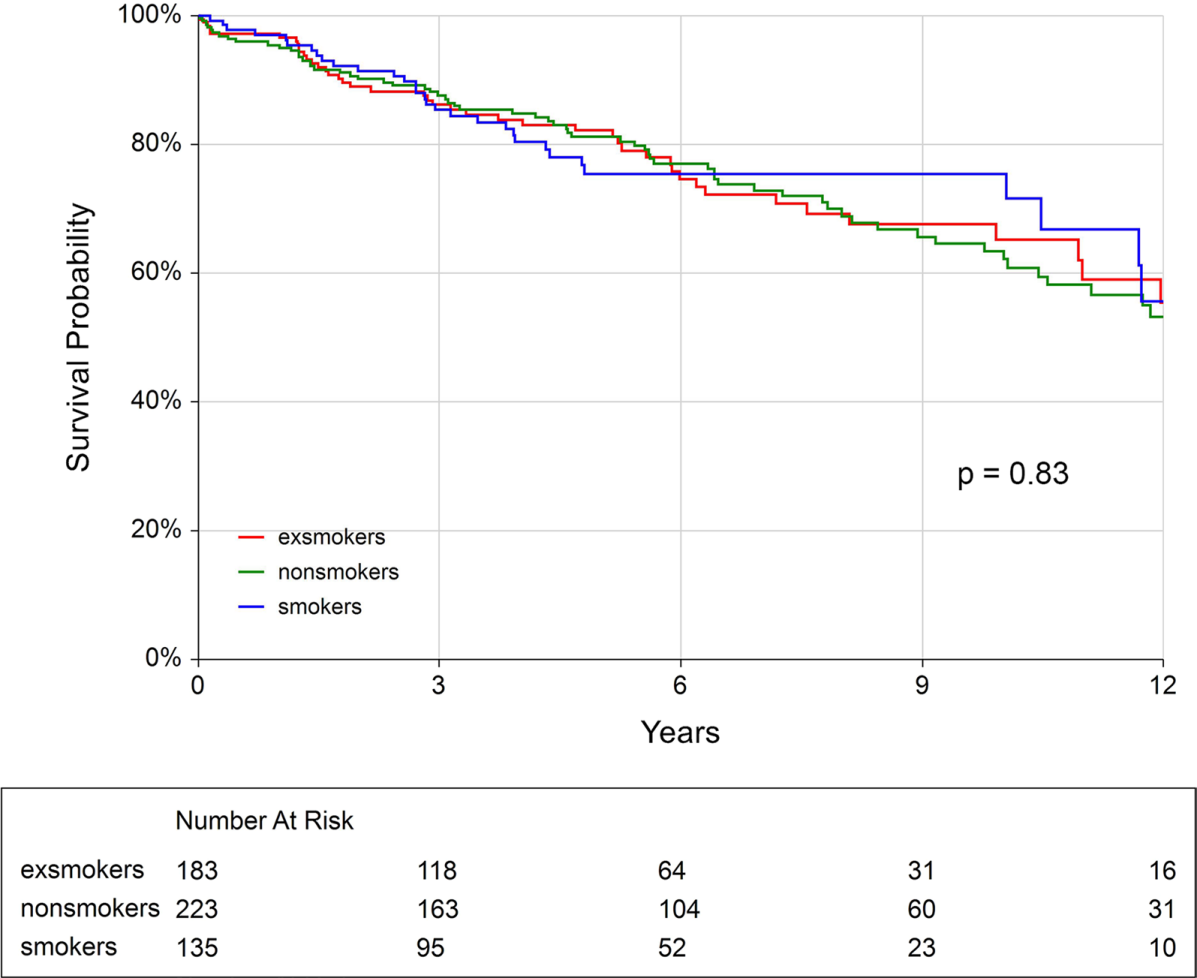
The Kaplan–Meier analysis of the long-term all-cause postoperative survival according to the smoking status

**Fig. 4:**
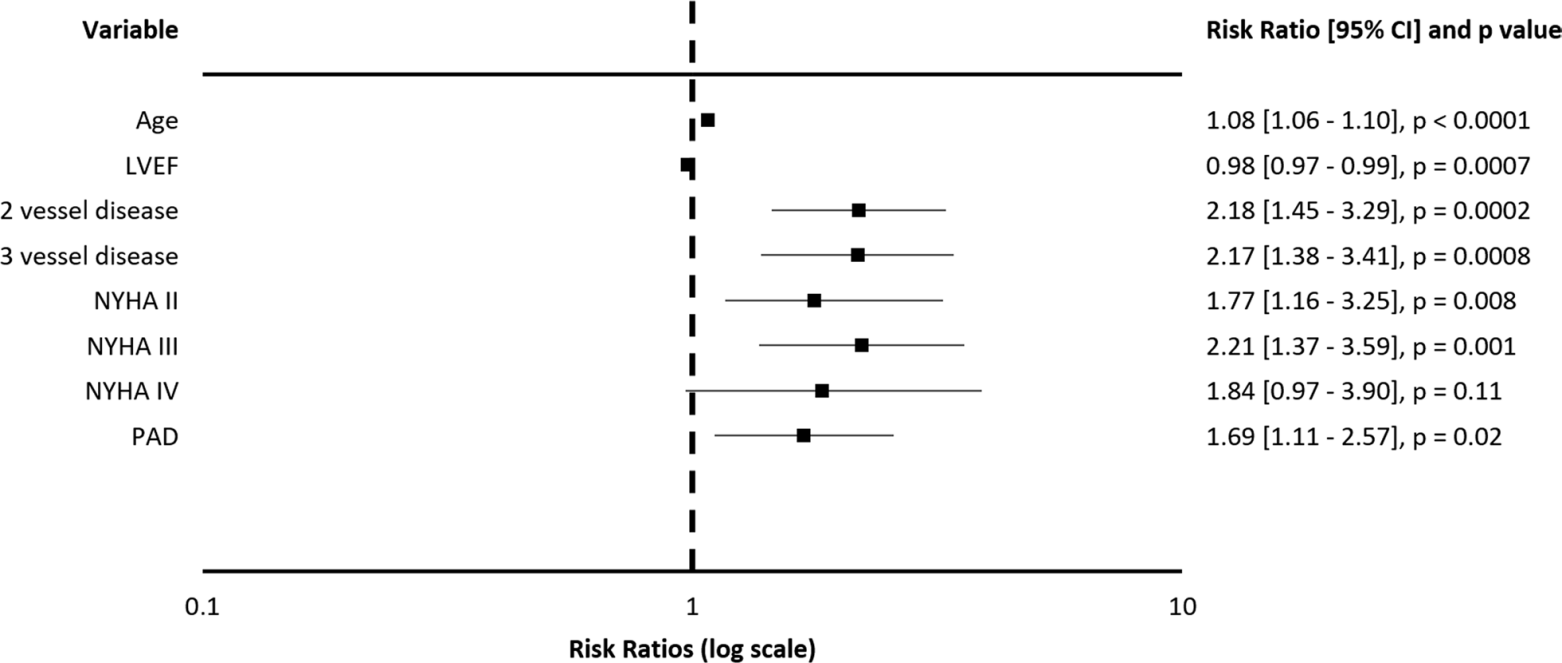
Multivariate Cox regression analysis for the long-term all-cause postoperative survival. Model R^2^ = 0.2811; Analysis of deviance for the whole model—p < 0.0001. CI: confidence interval; LVEF: left ventricular ejection fraction; NYHA: New York Heart Association classification of dyspnea; PAD: peripheral artery disease

Over the median follow-up of five years (up to 15 years), the all-cause postoperative mortality (see Fig. 3), as well as the composite of cardiac and cerebrovascular mortality only (p = 0.44, see Additional file 1: Figs. S5–6) were comparable among the study groups. The preoperative smoking status did not have any influence on the postoperative mortality in the long-term. This was rather influenced by the patient’s age, the systolic function of the left ventricle, the severity of coronary artery disease and other covariates (see Fig. 4).

## Discussion

Despite the abundant evidence in the pertinent literature on the impact of tobacco smoking on the short- and long-term outcomes of bypass surgery, studies regarding the outcomes of MIDCAB are lacking. In our retrospective study we found active smokers to present for MIDCAB at a younger age compared to non-smokers and ex-smokers. This coincides with the findings from previous studies performed on standard CABG [8, 12, 13]. Similarly to Al-Sarraf et al. [8], we also found that smokers were more likely to present for an urgent revascularization. Hypothetically, the long-term active smoking could lead to an accelerated formation of unstable atherosclerotic coronary plaques, consequently causing an acute coronary syndrome. As we expected, active smokers presented most frequently with a history of an atherosclerotic disorder (represented by peripheral arterial disease and myocardial infarction) in concordance with other studies [8, 12]. This may be attributed to the underlying pathomechanisms leading to accelerated atherosclerosis and hypertension [14–16] in smokers. Our multi-variate analysis yielded that treated hyperlipidemia was an independent protective factor against the need for an urgent revascularization. This corresponds to the findings of a previous study by Ji et al. [12] where treated hyperlipidemia was an independent protective factor against the need for an urgent revascularization. It might be due to the fact that an early diagnosed and treated hyperlipidemia reduces the risk of development of an acute coronary syndrome, as shown also by Alpe et al. [17] who studied the effect of preoperative administration of statins on cardiac surgery.

Our multi-variate analysis found active smokers to have the highest rate of post-operative pulmonary complications. This association was not found in ex-smokers which chimes with other studies [12, 13, 18–20]. It may be attributed to the ciliary dysfunction that arises in chronic smokers [21, 22]. Although we were unable to identify a cut-off time interval before surgery for smoking cessation to achieve its maximum benefit, Warner et al. [18] found that ex-smokers with a smoking cessation period greater than 6 months had a similar rate of postoperative pulmonary complications to non-smokers, and recommended a minimum period of 8 weeks of smoking cessation before undergoing coronary surgery to maximize the possible benefit. Other studies suggest that smoking cessation as early as possible regardless of the interval prior to surgery should be strongly recommended [7]. This is supported by the evidence that suggests a substantial microscopic enhancement in lung functions only weeks after smoking cessation and complete recovery after 6 months [23].

Although statistically insignificant, we found that non-smokers had that highest rate of conversions to sternotomies. However, this was not associated with the smoking status but was rather of a technical nature. Of the total 14 conversions accounted for in our study, 7 were due to injury of the LIMA during harvesting, 5 were due to suboptimal patient anatomy (extreme obesity; LIMA or LAD inaccessible through a thoracotomy), and 2 were due to injury to the right ventricle during the procedure.

Nonsmokers were most frequently associated with wound healing complications. However, this relationship was not found to be valid after the multivariate analysis. This was rather caused by the highest proportion of female patients being in the non-smokers group, as supported by previous evidence [24].

We did not find a correlation between the smoking status and the long-term post-operative mortality, accounting for either the all-cause mortality or only the composite of cardiac and cerebrovascular mortality. This is in contrast to other studies which found an increased rate of all-cause mortality in smokers, mainly attributable to cardiovascular causes [21]. He et al. [25] previously described even an increased 30-day and in-hospital post-operative mortality in smokers after cardiac surgery. This absence of mortality difference in our cohort might be due to constant improvement in perioperative care and reduction of mortality from respiratory causes. Our multivariate analysis yielded a few independent risk factors for long-term mortality, which included age, severe coronary artery disease, reduced ejection fraction, severity of heart failure and peripheral artery disease, similarly to another study performed by Domburg et al. [20]

### Limitations

This study was a retrospective observational trial, which may influence its generalizability. A low perioperative mortality rate did not allow us to further elaborate on this topic. This study focused on the early clinical outcomes and the long-term survival. We were unable to investigate the long-term risk of coronary re-interventions as we did not have access to these data for General Data Protection Regulation (GDPR) reasons. We did not have the information on the eventual postoperative smoking cessation that could also influence the long-term outcomes.

## Conclusions

Active smokers represent a specific cohort of patients undergoing coronary surgery. They present for MIDCAB at a younger age and with a more advanced atherosclerotic disorder. Tobacco smoking is associated with a greater need for an urgent surgery and is also the most significant risk factor for postoperative pulmonary complications. Long-term postoperative survival was not affected by the preoperative smoking status in our study, but was rather influenced by the patient’s age, left ventricular ejection fraction, severity of coronary artery disease and heart failure, and by the presence of peripheral artery disease.

## Supplementary Information

**Additional file 1. ** Supplementary Figures and Table.

## Data Availability

The datasets used and/or analyzed during the current study are available from the corresponding author on reasonable request. Long-term data on mortality were provided by the Institute of Health Information and Statistics of the Czech Republic (https://www.uzis.cz/), which is an official institution of the Czech Ministry of Health.

## Abbreviations

MIDCAB: Minimally invasive direct coronary artery bypass grafting
LAD: Left anterior descending artery
LIMA: Left internal mammary artery graft
COPD: Chronic obstructive pulmonary disease
