# Supplementary material for: Impact of smoking on the outcomes of minimally invasive direct coronary artery bypass
Source: J Cardiothorac Surg. 2023 Jan 20;18:43. doi: 10.1186/s13019-023-02104-9 (PMC9862783; doi:10.1186/s13019-023-02104-9)
Supplement: Supplementary file 1 — Additional file 1. Supplementary Figures and Table. [file 13019_2023_2104_MOESM1_ESM.docx]

**The Impact of Smoking Status**

**on the Outcomes of MIDCAB**

**Supplementary Material**

**
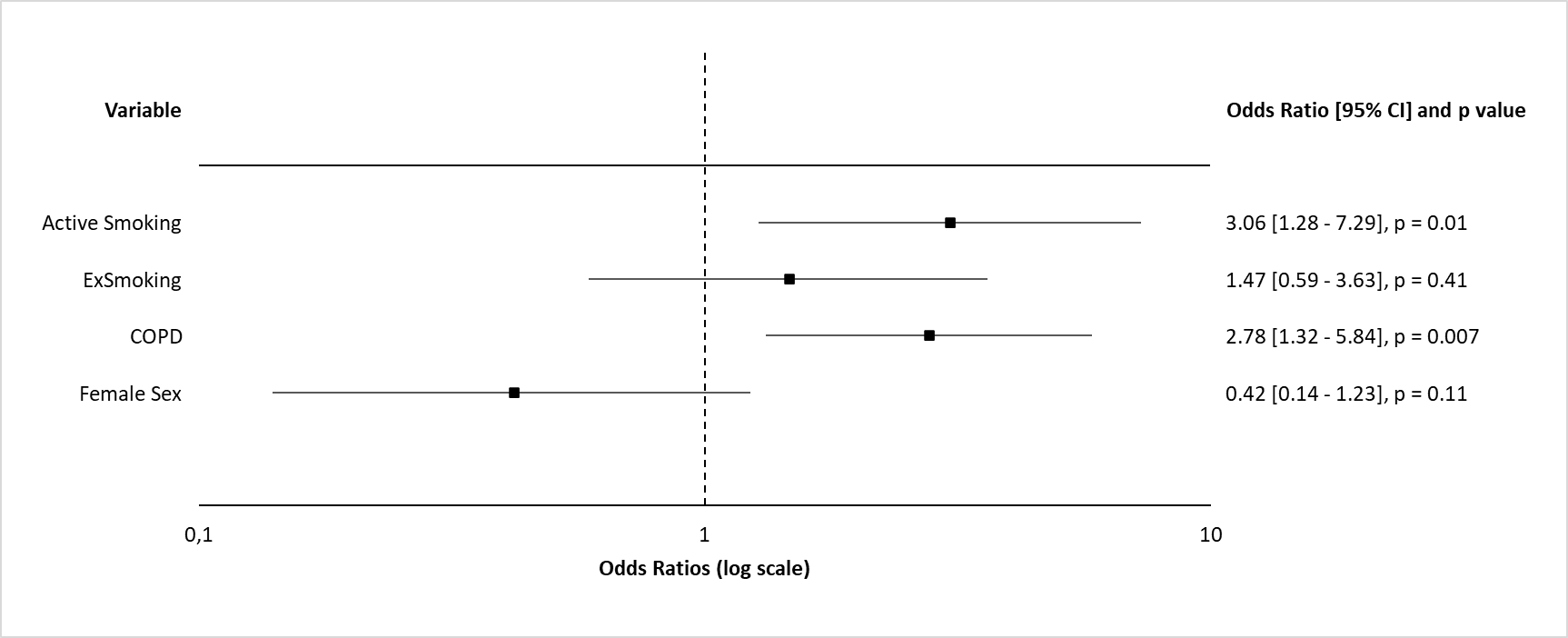
Supplementary figure 1 - Logistic regression analysis for the postoperative subcutaneous emphysema.**

*Model R^2^ = 0.0866; Analysis of deviance for the whole model - p < 0.0001.*

*CI - confidence interval; COPD - chronic obstructive pulmonary disease.*

**
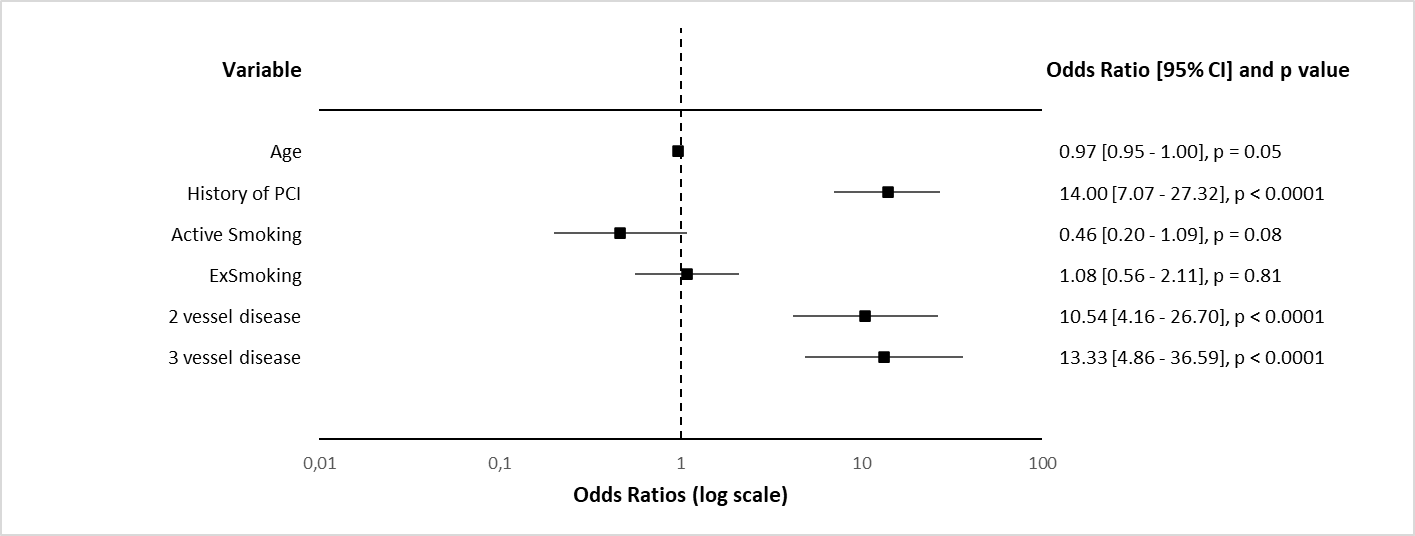
Supplementary figure 2 - Logistic regression analysis for the hybrid treatment strategy.**

*Model R^2^ = 0.3437; Analysis of deviance for the whole model - p < 0.0001.*

*CI - confidence interval; PCI - percutaneous coronary intervention.*

**
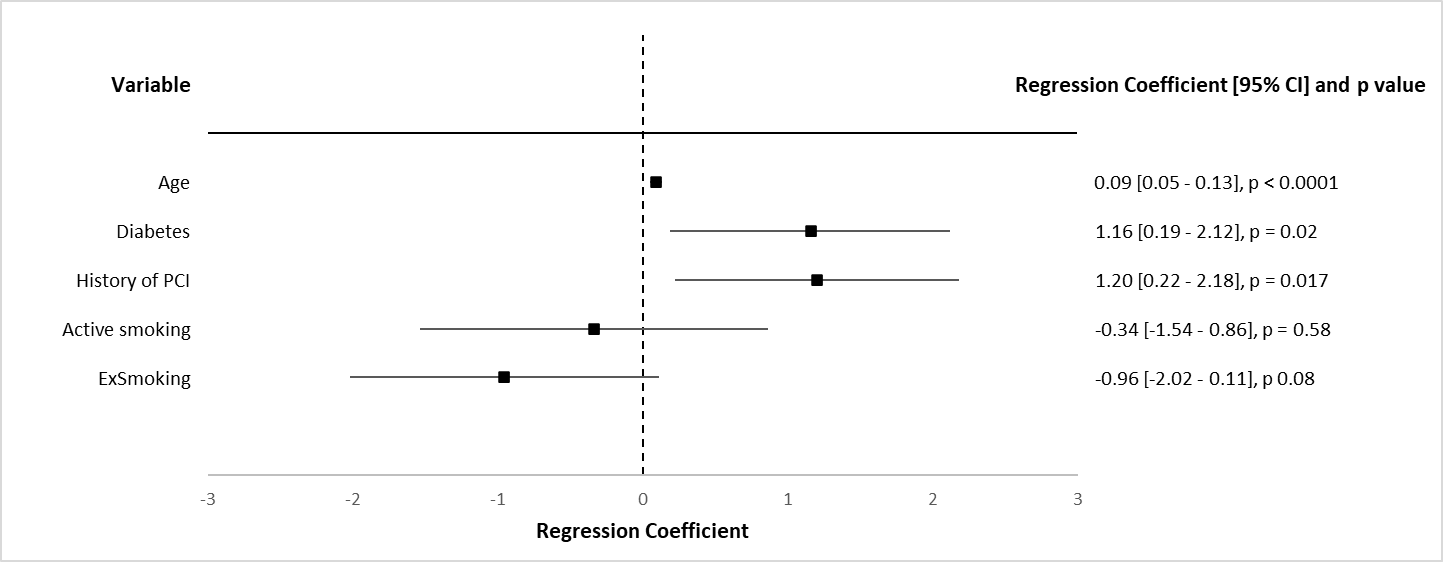
Supplementary figure 3 - Multiple regression analysis for the length of hospital stay.**

*Model R^2^ = 0.0675; Analysis of variance for the whole model - p < 0.0001.*

*CI - confidence interval; PCI - percutaneous coronary intervention.*

**Supplementary figure 4 - Logistic regression analysis for the risk of wound healing complications.**

**
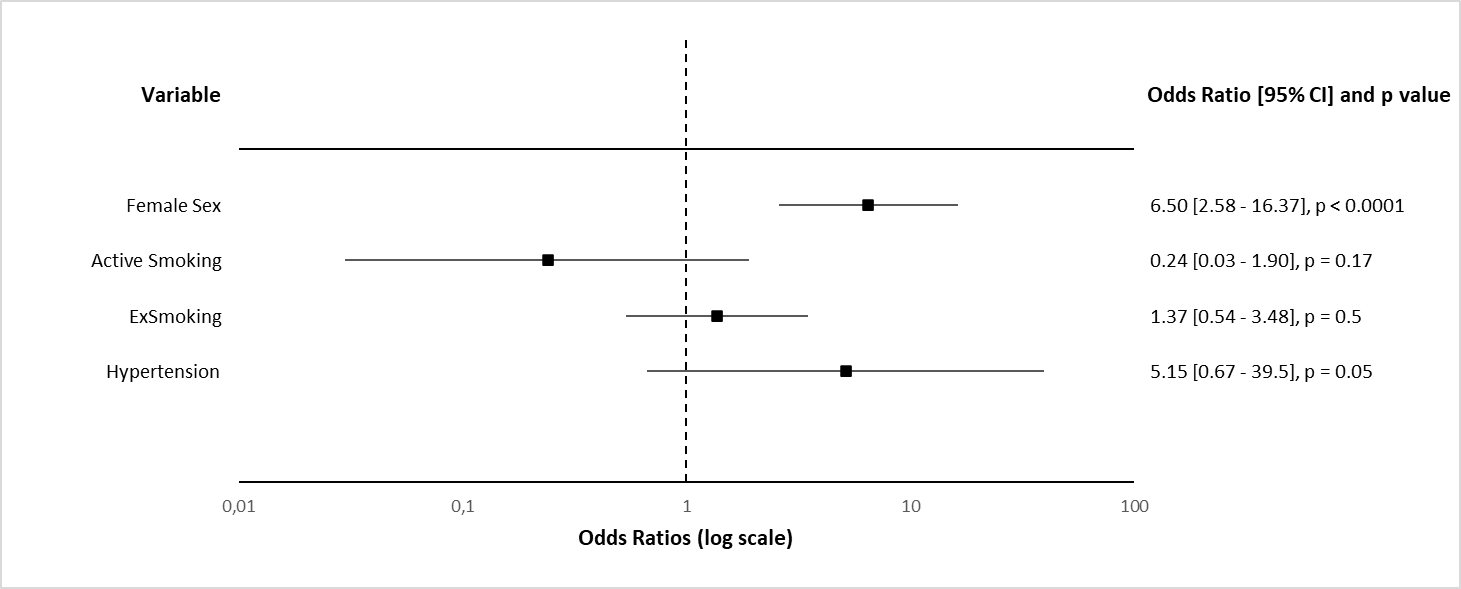
**

*Model R^2^ = 0.1557; Analysis of deviance for the whole model - p < 0.0001.*

**Supplementary figure 5 - Kaplan-Meier analysis of the composite of cardiac and cerebrovascular mortality in the long-term postoperatively according to the smoking status.**


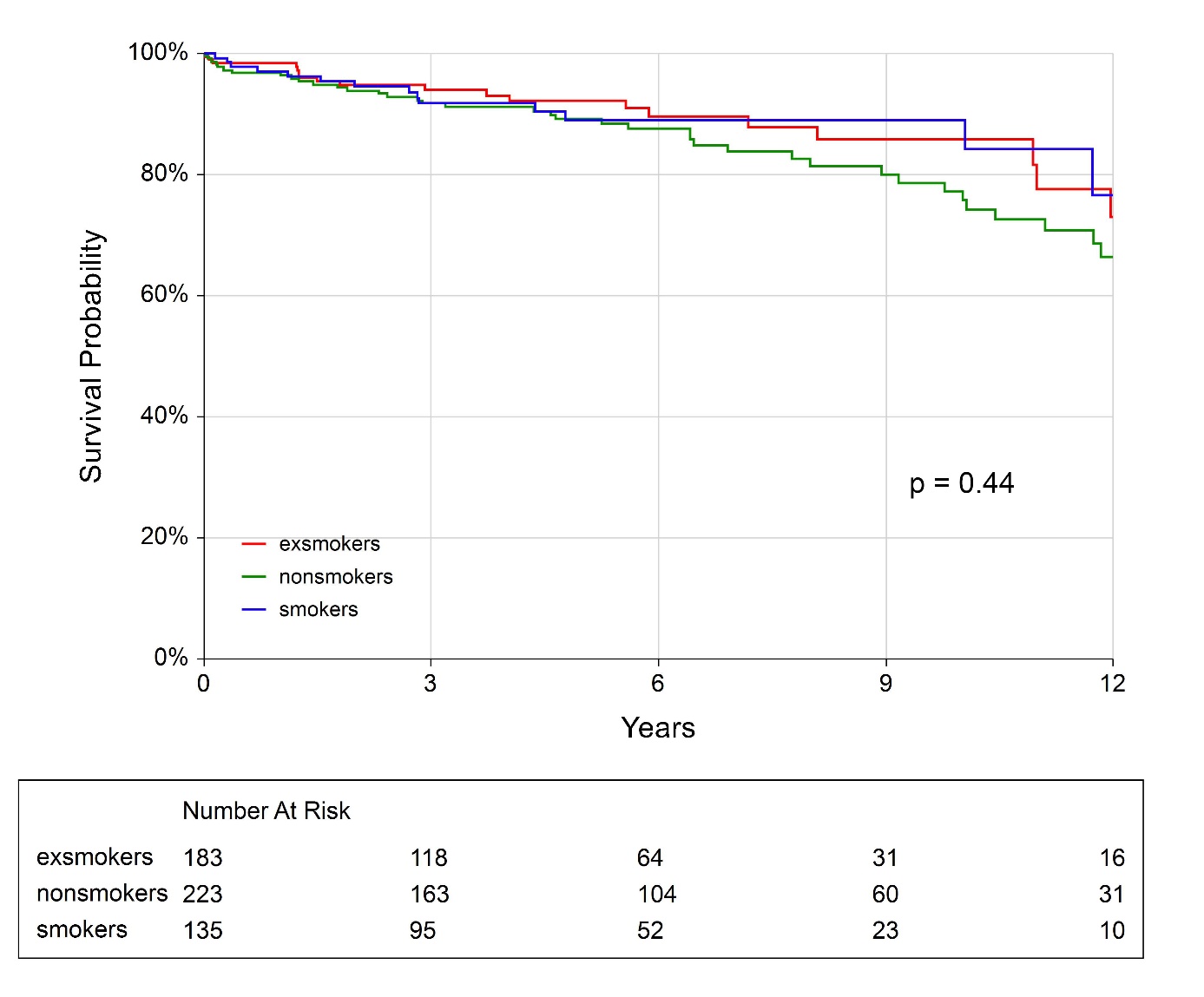


**Supplementary figure 6 - Multivariate Cox regression analysis for composite of cardiac and cerebrovascular mortality in the long-term postoperatively.**

**
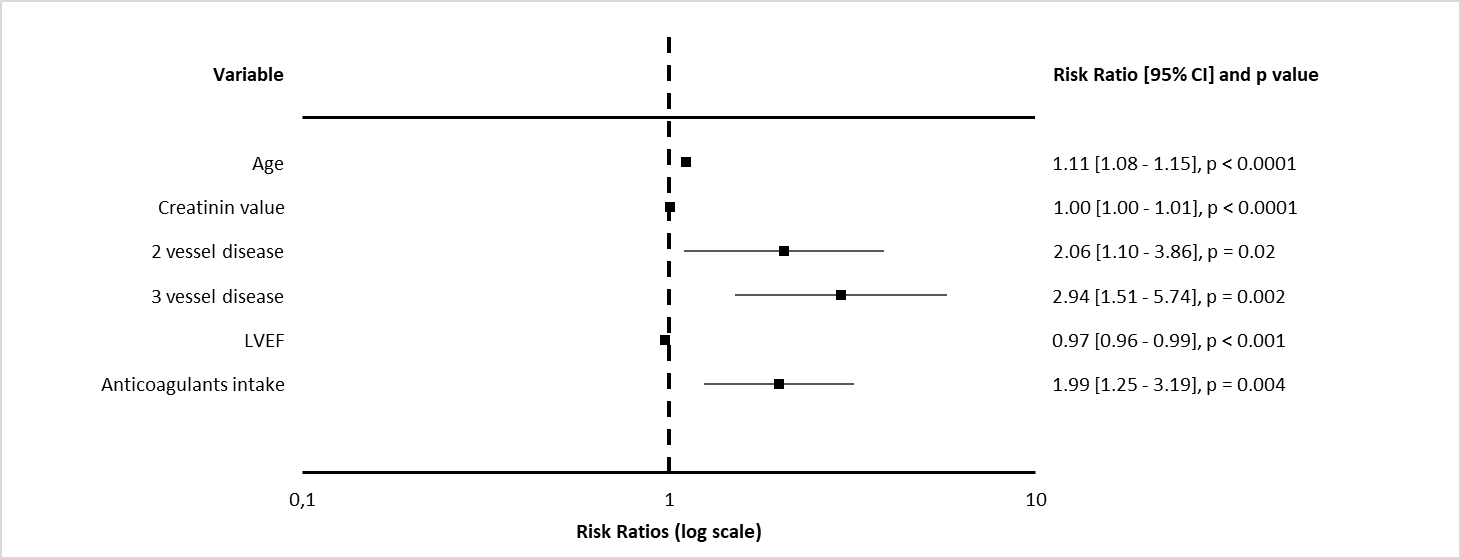
**

*Model R^2^ = 0.2196; Analysis of deviance for the whole model - p < 0.0001.*

**Supplementary Table 1 - Cut-off analysis for the preoperative smoking cessation timing with regard to the prevalence of the composite of postoperative pulmonary complications.**

| **Smoking cessation time preoperatively (months )** | **Prevalence of composite outcome under the cut-off (%)** | **Prevalence of composite outcome above the cut-off (%)** | **p value** |
| --- | --- | --- | --- |
| **6** | 18.6 | 17.4 | 0.86 |
| **12** | 19.6 | 16.8 | 0.64 |
| **24** | 18.5 | 17.2 | 0.84 |
| **36** | 17.7 | 17.7 | 0.99 |
| **60** | 17.6 | 17.8 | 0.97 |
| **120** | 20.0 | 15.1 | 0.39 |
| **180** | 19.5 | 14.3 | 0.38 |
| **240** | 19.0 | 12.8 | 0.37 |
